# Evaluating the effectiveness of a healthy lifestyle clinician in addressing the chronic disease risk behaviours of community mental health clients: study protocol for a randomised controlled trial

**DOI:** 10.1186/s13063-017-2017-1

**Published:** 2017-06-15

**Authors:** Caitlin Fehily, Kate Bartlem, John Wiggers, Paula Wye, Richard Clancy, David Castle, Sonia Wutzke, Chris Rissel, Andrew Wilson, Paul McCombie, Fionna Murphy, Jenny Bowman

**Affiliations:** 1 The University of Newcastle, Callaghan, NSW Australia; 2 The Australian Prevention Partnership Centre (TAPPC), Sax Institute, Ultimo, NSW Australia; 3 Population Health, Hunter New England Local Health District, Wallsend, NSW Australia; 4 Hunter Medical Research Institute, Clinical Research Centre, New Lambton Heights, Australia; 5 The University of Melbourne, Parkville, VIC Australia; 6 St Vincent’s Health, Fitzroy, VIC Australia; 7 NSW Office of Preventive Health, Liverpool, NSW Australia; 8 The University of Sydney, Sydney, NSW Australia; 9 Menzies Centre for Health Policy, University of Sydney, Sydney, NSW Australia; 10 Hunter New England Local Health District, NSW Health, New Lambton, NSW Australia

**Keywords:** Mental health, Physical health, Community mental health, Mental health services, Smoking, Nutrition, Alcohol, Physical activity

## Abstract

**Background:**

People with a mental illness experience a greater morbidity and mortality from chronic diseases relative to the general population. A higher prevalence of modifiable health risk behaviours such as smoking, poor nutrition, physical inactivity and harmful alcohol consumption contribute substantially to this disparity. Despite clinical practice guidelines recommending that mental health services routinely provide care to address these risk behaviours, the provision of such care is consistently reported to be low internationally and in Australia. This protocol describes a randomised controlled trial that aims to assess the effectiveness of allocating a clinician within a community mental health service to the specific role of providing assessment, advice and referral for clients’ chronic disease risk behaviours.

**Methods/design:**

Approximately 540 clients of one community mental health service will be randomised to receive either usual care for chronic disease risks provided in routine consultations or usual care plus an additional face-to-face consultation and follow-up telephone call with a ‘healthy lifestyle clinician’. The clinician will assess clients’ chronic disease risk behaviours, provide advice to change behaviours, and refer at-risk clients to free telephone coaching services (New South Wales (NSW) Quitline and NSW Get Healthy Information and Coaching Service) for specialist behaviour change care. The primary outcomes, regarding referral to and client uptake of the telephone services, will be obtained from the respective services. Telephone interviews of clients at baseline and at 1 and 6 months post baseline follow-ups will assess secondary outcomes: receipt of any assessment, advice and referral from the mental health service; satisfaction with the receipt of such care; satisfaction with the receipt of any care provided by the telephone services; interest and confidence in and perceived importance of changing risk behaviours; and risk behaviour status.

**Discussion:**

This study will add to the limited literature regarding effective strategies to address chronic disease prevention among the higher risk population of community mental health clients. The results will inform the development of future policies and service delivery initiatives to address the high prevalence of chronic disease risk behaviours among people with a mental illness.

**Trial registration:**

Australian New Zealand Clinical Trials Registry (ANZCTR), ACTRN12616001519448. Registered on 3 November 2016.

**Electronic supplementary material:**

The online version of this article (doi:10.1186/s13063-017-2017-1) contains supplementary material, which is available to authorized users.

## Background

People with a mental illness experience a greater morbidity and mortality from chronic diseases relative to the general population, and this disparity is even greater for people with severe mental illness [1–4]. In Australia, the gap in life expectancy for people with a mental illness is estimated to be 16 years for males and 12 years for females, with approximately 78% of this excess death being attributable to physical health conditions such as cardiovascular disease, diabetes and cancer [5]. A higher prevalence of modifiable health risk behaviours, including smoking, poor nutrition, harmful alcohol consumption and inadequate physical activity [6–8], contribute to this chronic disease burden [7, 9]. For example, smoking rates among people with a mental illness are up to three times higher than that of the general population in Australia [10–12], the UK [13] and the USA [14]. Moreover, risk for two or more chronic disease risk behaviours was present in 78% of clients of Australian community mental health services [15], compared to 31% of the general population [16].

Clinical practice guidelines recommend that chronic disease risk behaviours be routinely assessed and managed by health care services [17–19], including specialist mental health services [20–22]. As a minimum, it has been suggested that this care may follow a ‘2As and an R’ approach: *assessment* of clients’ risk for chronic disease behaviours, provision of brief *advice* to modify behaviours and *referral* of clients to support services for ongoing specialist care [23–25].

Despite such guidelines, the provision of care by mental health clinicians to modify chronic disease risk behaviours is consistently reported to be sub-optimal in Australia [11, 26, 27] and internationally [28–30]. For example, a survey of Australian community mental health services found that few clinicians reported the provision of care for four key chronic disease risk behaviours (smoking, inadequate fruit and vegetable consumption, harmful alcohol consumption and physical inactivity), with only 9% reporting assessing most (at least 80% of) clients for all such risks [26]. Furthermore, where risks were identified, only 25% of respondents stated that they were offering most at-risk clients brief advice, and 10% were referring most at-risk clients to specialist behaviour change services to assist clients in modifying their chronic disease risk behaviours. In the USA, a survey of community mental health staff in one service (*n* = 154) found that only 27% reported that they provided advice to more than half of their clients regarding smoking, inadequate physical activity and insufficient fruit and vegetable consumption [28]. Such a failure to provide preventive care has been suggested to be a further contributor to the chronic disease burden experienced by people with a mental illness [31, 32]. Reasons for clinicians not addressing chronic disease risk behaviours in mental health settings have been suggested to include inadequate time and resources, lack of clinician training and low clinician confidence regarding the provision of such care [33, 34].

One approach to overcoming barriers to the provision of preventive care which has some support with mental health professionals and clients [35, 36] is to allocate a clinician to the specialist role of providing such care in the context of mental health care delivery [37–39]. Two randomised controlled trials within mental health settings have assessed the efficacy of specialist roles in increasing the provision of care to address chronic disease risks, including at least one behavioural risk [38, 39]. Osborn and colleagues [39] conducted a cluster randomised controlled trial of six mental health services in the UK to examine the efficacy of allocating a nurse to deliver a 6 month intervention to increase assessment of cardiovascular risks (tobacco smoking and biomedical risks such as high blood pressure and cholesterol). The nurse’s role was to (1) promote cardiovascular screening in routine mental health consultations by mental health clinicians by establishing monitoring systems for risk assessment and the use of reminders, and (2) provide cardiovascular screening to those clients who did not receive screening during their routine mental health consultations. Following the 6 month intervention, rates of cardiovascular risk screening increased by at least 30% for each risk, compared to mental health services that were provided with an educational package regarding the provision of such care only. No significant difference was found between groups in the proportion of clients receiving a referral to smoking cessation support services in the nurse-led intervention (49%) compared with the control condition (49%).

In the USA, Druss and colleagues [38] undertook a randomised controlled trial (*n* = 407) to assess the potential efficacy of a care management model to increase access to primary and preventive care services. The intervention involved a ‘medical care nurse’ located in a single community mental health service whose primary role was to assist clients in overcoming barriers to accessing primary medical care. The nurse provided chronic disease health education, advice to support health risk behaviour change, liaison with primary care providers on behalf of clients and support for clients in contacting primary care providers. The impact of the intervention was measured in terms of an aggregate score of services the client received regarding their physical health, including education regarding chronic disease risk behaviours, physical examination, screening tests and vaccinations. At a 12 months follow-up, the intervention group received a significantly higher proportion of such services than those who received usual care (58.7% vs 21.8% respectively). No results were reported regarding care addressing chronic disease risk behaviours specifically.

Further research is required to determine the effectiveness of embedding a specialist care provider in a community mental health setting with the role of providing assessment, advice and referral for multiple behavioural chronic disease risks. To address this limited evidence, a randomised controlled trial will be conducted to assess the effectiveness of a ‘healthy lifestyle clinician’ located in a community mental health service in increasing the provision of assessment, advice and referral for four key chronic disease risk behaviours: smoking, poor nutrition, physical inactivity and harmful alcohol consumption.

## Methods/design

### Study design and setting

**Fig. 1:**
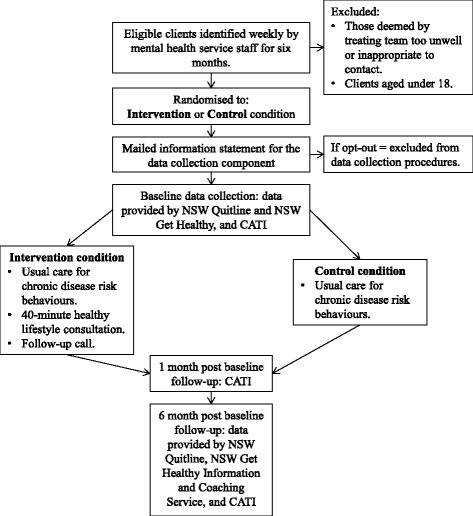
Study design flow diagram. *CATI* computer-assisted telephone interview

**Fig. 2:**
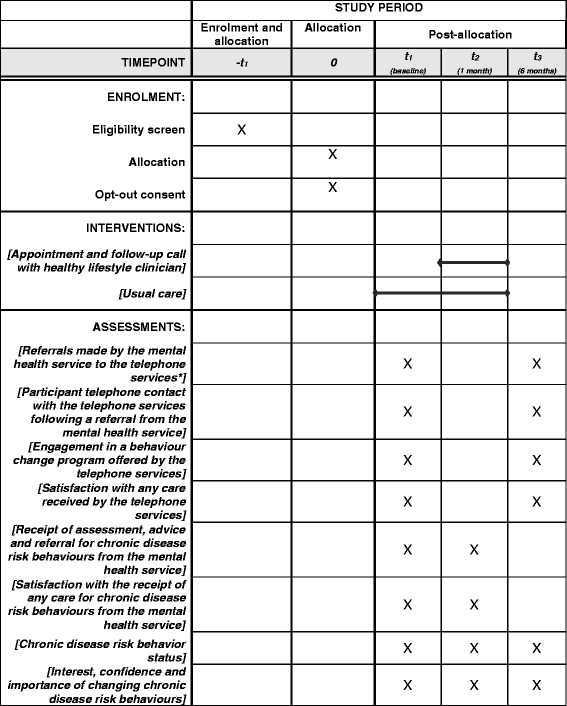
SPIRIT schedule of enrolment, intervention and assessments. *Telephone services = New South Wales (*NSW*) Quitline and NSW Get Healthy Information and Coaching Service

A parallel-group randomised controlled trial with blinded follow-up will be undertaken (see Fig. 1 for the study flow diagram, Fig. 2 for the Standard Protocol Items: Recommendations for Interventional Trials (SPIRIT) diagram and Additional file 1 for the SPIRIT checklist). The study will be conducted in one community-based mental health service in a regional area in the state of New South Wales (NSW), Australia. The service is typical of regional community-based mental health services, seeing clients with a range of mental health issues (such as depression, psychoses, anxiety and bipolar disorder) and severities. In 2010, the health district within which the service is located implemented a policy requiring clinician assessment, advice and referral to ongoing behaviour change support services for clients with chronic disease risk behaviours, including smoking, inadequate fruit and vegetable consumption, physical inactivity and harmful alcohol consumption [40]. Rates of adherence to this policy in community mental health services have been reported to be sub-optimal [26].

The community mental health service is implementing a new approach to meeting the policy requirements, whereby a healthy lifestyle clinician will be embedded within the service for a period of 6 months, with the specific role of providing assessment, advice and referral to evidence-based behaviour change services to modify the chronic disease risk behaviours of clients. The purpose of this trial is to evaluate this approach to care provision. Clients will be randomly allocated to receive usual care (including any care for chronic disease risk behaviours that might be provided in the context of routine mental health treatment) or usual care plus an additional face-to-face consultation and follow-up call with the healthy lifestyle clinician. The primary purpose of the additional care component will be to identify at-risk clients and provide motivation for modification of the behaviour and uptake of free government-provided telephone coaching services (NSW Quitline (smoking) and NSW Get Healthy Information and Coaching Service (nutrition, physical activity, alcohol consumption and weight management)). Primary outcome data will be obtained from the respective services at baseline and at 6 months post baseline and will include (1) referrals made by the community mental health service to the telephone services, (2) client contact with the telephone services and (3) client engagement in a behaviour change program offered by the telephone services. Secondary outcomes will be client-reported receipt of any assessment, advice and referral for chronic disease risk behaviours from the mental health service; satisfaction with the receipt of such care; satisfaction with the receipt of any care provided by the telephone services; interest and confidence in and importance of changing chronic disease risk behaviours; and risk behaviour status. Secondary outcome data will be collected by computer-assisted telephone interviews (CATIs) undertaken with clients at baseline and at 1 and 6 months post baseline follow-ups. The effectiveness of the healthy lifestyle clinician role will be assessed by comparing primary and secondary outcomes between the intervention and control conditions at follow-up.

The trial has been approved by the Hunter New England Human Research Ethics Committee (Ref no. 16/02/17/4.09) and the University of Newcastle Human Research Ethics Committee (Ref no. H-2016-0123).

### Participants

Participants will be all clients of one community mental health service in Australia. Community mental health staff will identify on a weekly basis all new and existing clients of the service during a 6 month period. Clients who are under the age of 18 or identified by mental health staff to be too unwell or clinically inappropriate to contact will be ineligible for inclusion. Eligible clients will be mailed a study information statement explaining the telephone interviews and data collection procedures. Clients will be provided a toll-free number to call should they not wish to participate in data collection. This procedure is based on those previously used successfully in this setting [41]. Participation in data collection will be independent of care that may be provided by usual clinicians and/or the healthy lifestyle clinician.

### Randomisation

Prior to commencement of the trial, a random allocation sequence using permuted-block randomisation (with block sizes of 2, 4 and 6) will be generated by a statistician independent of the project. Eligible clients will be randomly allocated in a 1:1 ratio to receive usual care (control condition) or usual care plus a consultation and follow-up call with the healthy lifestyle clinician (intervention condition). Clients will not be blinded to the study allocation.

### Intervention condition

Clients allocated to the intervention condition will be notified by a mailed letter which will briefly describe the additional care they will receive. They will receive a 40-minute consultation and follow-up call with the healthy lifestyle clinician, in addition to care they might receive in the context of their routine mental health consultations. The healthy lifestyle clinician will phone clients to arrange the additional consultation to occur either following an existing mental health consultation appointment or as a standalone appointment.

The healthy lifestyle clinician will be embedded as a member of the community mental health service for the duration of the trial. The clinician will have prior experience working with individuals with a mental illness and will undergo additional training in the delivery of the intervention.

The intervention will be implemented according to a manualised protocol based on motivational interviewing principles [42, 43], with the aims of fostering client motivation for modification of risk behaviours and uptake of telephone coaching services (NSW Quitline (smoking) and NSW Get Healthy Information and Coaching Service (poor nutrition, physical inactivity, alcohol consumption and weight management)). In line with the physical health care policy of the service [40], the manual will guide the healthy lifestyle clinician to:

1. Assess risk: The healthy lifestyle clinician will assess the client’s chronic disease risk behaviours through a structured questionnaire evaluating the client’s risk status in accordance with national guidelines. Risk is defined as follows: any tobacco smoking [44]; consuming more than two standard drinks on an average day or more than four standard drinks on any one occasion [45]; consuming less than two serves of fruit or five serves of vegetables daily as an indicator of poor nutrition [46]; or engaging in less than 150 minutes of moderate intensity physical activity or 75 minutes of vigorous intensity physical activity, or an equivalent combination each week [47]. The clinician will assess abdominal adiposity as a sequela of physical inactivity and inadequate fruit and vegetable consumption, where risk is defined as abdominal adiposity (waist circumference) being ≥94 cm in males or ≥80 cm in females [48]. The clinician will also ask the client if he/she has any concerns in relation to weight or nutrition more broadly than fruit and vegetable consumption.
2. Advise: The clinician will provide brief feedback to the client in a non-judgemental manner as to how his/her behaviours compare against national guidelines. Clients identified as meeting the national guidelines for one or more of the behaviours will be provided positive feedback on the health benefits of their behaviour. If the client is identified as being at risk according to the national guidelines for any one or more of the behaviours, the healthy lifestyle clinician will provide brief advice to modify the behaviour. Further intervention based on motivational interviewing techniques [42, 43] will be provided for the risk behaviours, which is designed to address any ambivalence regarding the current behaviour and motivate for change. Specific strategies utilised and the content discussed will be tailored to each client and may include education about current levels of behaviour and associated health impacts; further information about guidelines and recommendations reinforcing positive intentions to change behaviour; consideration of the barriers to change; and building self-efficacy [42, 43].
3. Refer: Clients who are at risk for smoking will be offered a referral to the NSW Quitline (for more information, access https://www.icanquit.com.au/further-resources/quitline). Clients who are at risk and/or express concern about their physical activity, weight, nutrition or alcohol consumption will be offered a referral to the NSW Get Healthy Information and Coaching Service (for more information, access http://www.gethealthynsw.com.au/). If appropriate, clients will be offered a referral to both telephone services. The NSW Get Healthy Information and Coaching Service offers ten free individual coaching sessions over a 6 month period to support individuals in setting and achieving weight-related behaviour change goals [49]. The NSW Quitline offers six free individual telephone coaching sessions to support individuals to stop smoking [50]. In line with clinical practice guidelines, all clients will be referred to their general practitioner (GP) for assessment of metabolic risks [22].

Approximately 1 week following the consultation with the healthy lifestyle clinician, the clinician will call the client to follow up on any referrals made and briefly discuss his/her progress. Positive feedback will be provided to clients who have had contact with the telephone services and/or attended the appointment with their GP, to further encourage behaviour change. Where clients have not yet taken up referrals made, the clinician will provide encouragement to do so and seek to identify and address any barriers the client may have.

### Control condition

Those allocated to the control condition will receive care for chronic disease risk behaviours as is normally provided in routine consultations. In line with the current policy of the health district, this care should include assessment of chronic disease risk behaviours (including smoking, fruit and vegetable consumption, physical activity and alcohol use), brief advice relating to the health behaviour, offer of referral to specialist behaviour change services as appropriate [40] and referral to a GP for assessment and management of metabolic risks [51].

### Data collection procedures

The data collection procedures and measures are summarised in Table 1. For participating clients, socio-demographic and clinical information will be obtained from electronic service records, including contact details, primary mental health diagnosis, date commenced as a client of the service and the number of appointments attended. Additional characteristics will be collected during the baseline CATI, including age, gender, highest level of education completed, employment status, current marital status and Aboriginal and/or Torres Strait Islander status.

**Table 1:** Data collection procedures and measures

Data source	Data collected	Time points		
		Baseline	1 month	6 months
NSW Quitline	Referrals received from the community mental health service	✓	-	✓
	Any telephone contact with the telephone service in the last 6 months	✓	-	✓
	Acceptance of at least one follow-up coaching call from the telephone service in the last 6 months	✓	-	✓
NSW Get Healthy Information and Coaching Service	Referrals received from the community mental health service	✓	-	✓
	Any telephone contact with the telephone service in the last 6 months	✓	-	✓
	Enrolment in a ten-session behaviour change program in the last 6 months	✓	-	✓
CATI	Satisfaction with the receipt of any care provided by the NSW Quitline and/or NSW Get Healthy Information and Coaching service	✓	-	✓
	Chronic disease risk behaviour status in the last month	✓	✓	✓
	Interest, confidence and belief in importance of changing risk behaviours	✓	✓	✓
	Receipt of any assessment, advice and/or referral for risk behaviours from the mental health service in the last month	✓	✓	-
	Satisfaction with the receipt of any assessment, advice and/or referral for risk behaviours from the mental health service in the last month	✓	✓	-

*NSW* New South Wales, *CATI* computer-assisted telephone interview

Data regarding the primary outcome, referrals by the community mental health service to the NSW Quitline and NSW Get Healthy and Information Coaching Service and uptake of referrals will be obtained from the respective services at baseline and at 6 months post baseline follow-up, in relation to the previous 6 months. Data regarding the secondary outcomes will be obtained via CATI at baseline and at 1 month and 6 months post baseline follow-ups. The interview schedule will be based on previous research regarding care for chronic disease risk behaviours in mental health settings [11, 15, 41]. Participants will receive a reminder text message from the research team 3 days prior to each follow-up interview.

### Measures

#### Primary outcomes

The primary outcomes will be (1) referrals made by the community mental health service to the NSW Quitline and NSW Get Healthy Information and Coaching Service, (2) any telephone contact with the NSW Quitline and NSW Get Healthy Information and Coaching Service that is initiated by the telephone service following a referral from the community mental health service and (3) engagement in a behaviour change program offered by the telephone service. For the NSW Get Healthy Information and Coaching Service, this includes enrolling in the 6 month coaching program. For the NSW Quitline, this includes accepting at least one follow-up coaching call from the service.

#### Secondary outcomes

Secondary outcomes will include receipt of care for chronic disease risk behaviours from the mental health service; satisfaction with the receipt of such care; satisfaction with the receipt of any care provided by the telephone services; interest and confidence in and perceived importance of changing chronic disease risk behaviours; and risk behaviour status.

##### Receipt of care for chronic disease risk behaviours

At baseline and at the 1 month follow-up, participants will be asked to report their receipt of care for chronic disease risk behaviours from the mental health service in the previous month. In line with the local area health policy [40], this will include:

1. Assessment: Whether during an appointment at the community mental health service in the last month (with their usual clinician or the healthy lifestyle clinician) they were asked about their smoking status, fruit and vegetable consumption, alcohol consumption and physical activity (yes; no; don’t know)
2. Advice: Whether the community mental health service advised them to modify any of their health behaviours in the last month
3. Referral: Whether the community mental health service offered them a referral to the NSW Quitline and/or NSW Get Healthy Information and Coaching Service in the last month, and whether or not they accepted this referral. In addition, whether they were offered a referral to their GP for assessment of their metabolic risks and whether or not they accepted this referral

##### Interest and confidence in and perceived importance of changing chronic disease risk behaviours

Participants will be asked to report on a 1-to-10 scale the importance of changing and their confidence to make positive changes to each risk behaviour if they wish to do so. Participants will then be asked if they are seriously thinking about making any changes to their smoking, and if so, whether they have any plans for when they will make those changes (yes, in the next month; yes, in the next 6 months; yes, not in the next 6 months; not planning; don’t know).

##### Chronic disease risk behaviour status

Participants will be asked during each interview to report on their chronic disease risk behaviours, specifically:

1. Whether they smoked any tobacco products in the last month (yes, daily; yes, at least once a week; yes, less than once a week; not at all, quit less than 6 months ago; not at all, quit 6 months or more ago; not at all, never smoked)
2. How many serves of fruit (0, 1, 2 or more) and vegetables (0, 1, 2, 3, 4, 5, 6 or more) they usually ate per day in the last month
3. How often they had a drink containing alcohol in the last month (never, don’t drink; none in the last month; once; 2 to 4 times a month; 2 to 3 times a week; 4 or more times a week). Those who report having consumed alcohol during the month prior to the interview will be asked how many standard drinks they consumed on a typical drinking day (1, 2, 3, 4, 5, 6, 7, 8, 9, 10 or more) and how often they consumed more than four standard drinks on one occasion (never; less than monthly; monthly; weekly; daily or almost daily)
4. How much time they engaged in walking, moderate physical activity and vigorous physical activity [52], and how much time on average they spent in sedentary activities [53] each week over the last month

##### Process measures

During the baseline and 1 month follow-up interviews, participants will be asked to respond on a five-point Likert scale (‘not at all satisfied’ to ‘very satisfied’) regarding their satisfaction with each element of the chronic disease risk behaviour care provided by the mental health service. For example, one question is: How satisfied are you with the assessment of your health behaviours that you received from the mental health service?

Participants who self-reported contact with the NSW Quitline and/or NSW Get Healthy Information and Coaching Service will be asked to respond on a five-point Likert scale how satisfied they were with the care provided by the telephones service(s) (responses range from ‘not at all satisfied’ to ‘extremely satisfied’). In addition, participants will be asked whether they found the telephone service(s) to be helpful (‘not at all’ to ‘very much’) and if they would use the service(s) again if they wanted to make any behaviour changes in the future.

### Sample size

It is anticipated based on service patient throughput data that there will be approximately 750 eligible clients during the 6 month period (including both existing and new clients). Based on previous research in this setting it is estimated that 80% of these clients will consent to participate in the study [41], providing 540 clients for randomisation. It is estimated that approximately 50% of these clients will meet the eligibility criteria for a referral to the NSW Quitline and 90% will meet the criteria for a referral to the NSW Get Healthy Information and Coaching Service [15]. This sample will provide 80% power to detect an 8.9% difference in the proportion of clients who take up a referral to the NSW Get Healthy Information and Coaching Service and a 12.5% difference in the proportion smokers who take up a referral to the Quitline.

### Statistical analysis

Descriptive statistics will be used to describe client characteristics and assess for differences between the intervention and usual care conditions in terms of baseline characteristics. Logistic regression models will be used to compare the primary outcomes between the intervention and usual care conditions at the 6 months follow-up point, adjusting for baseline use of the telephone services and other potential confounders such as age, gender and number of visits to the mental health service. Similar analyses will be used for secondary outcomes. Analyses will be conducted on an intention-to-treat basis.

## Discussion

This study will add to the limited literature regarding the impact of a specialist role in a mental health setting to address chronic disease risk behaviours. The knowledge gained from this study will inform the development of future policies and service delivery initiatives to increase care provision for chronic disease risk behaviours. If shown to be effective, the uptake of the healthy lifestyle clinician role by community mental health services has the potential to have a positive impact on the chronic disease-related care that clients receive. Ultimately, this could reduce the disproportionately high prevalence of risk behaviours in this population and positively affect life expectancy and quality of life.

### Trial status

At the time of manuscript submission, participant recruitment had not yet commenced.

## Additional file

Additional file 1: SPIRIT checklist. (DOC 122 kb)
